# Relative Entropy Computations for Nonlinear Deformations of the Porous Steel Structures

**DOI:** 10.3390/ma19091783

**Published:** 2026-04-28

**Authors:** Michał Strąkowski, Marcin Kamiński

**Affiliations:** Department of Structural Mechanics, Faculty of Civil Engineering, Architecture and Environmental Engineering, Łódź University of Technology, 90-924 Łódź, Poland; michal.strakowski@p.lodz.pl

**Keywords:** relative entropy, stochastic finite element method, steel microdefects, Gurson-Tvergaard-Needleman model, yield surface, safety assessment

## Abstract

In this paper, we investigate the application of the relative entropy framework for safety assessments of steel elements with structural defects at the micro- and macro-scales. Mathematical theories developed by Bhattacharyya and by Kullback and Leibler (K-L) have been used for this purpose. This approach uses both expectations and variations, similar to the First-Order Reliability Method (FORM), but is extended to include 3rd- and 4th-order central probabilistic moments. It is necessary to use a hybrid computational technique that combines the Finite Element Method (FEM) software ABAQUS CAE 2017 with the implemented Gurson–Tvergaard–Needleman (GTN) damage model and the computer algebra system MAPLE. The iterative generalized stochastic perturbation technique has been used to determine the probabilistic moments of structural response, to utilize the Weighted Least Squares Method to approximate the structural response function, and to determine uncertainty in the stress, strain, and displacement state functions. This approach is based on relative entropy because of its universality. There is no need to assume a type of distribution of the state functions, in contrast to FORM, where a Gaussian distribution is required. This paper verifies whether relative entropy can serve as an alternative to FORM for determining reliability. The yield surface of the porous material with a random values of the void volume fraction *f* are also presented.

## 1. Introduction

The well-known Huber-Mises-Hencky strength hypothesis is not enough to analyze plasticized materials (including necking and large strains). The reason is the assumption of a material continuum, which ignores microstructural defects and their influence on material strength. Such a phenomenon requires a more advanced, accurate material model that accounts for material damage. There are a few constitutive theories with structural defects [1] and the Gurson model [2,3], with its improvements known by the surnames of Tvergaard and Needleman, is a material model frequently applied in computer analysis [4]. The central assumption is that micro-voids are disordered, spherical, with a minimal radius compared to the whole element’s dimensions. Based on experimentation and engineering intuition, the voids must exhibit uncertainty in growth, nucleation, and formation, and their initiation should be stochastic. A wide range of numerical techniques (Monte Carlo simulation [5], Bayesian methods [6], spectral methods [7], and stochastic perturbation methods [8]) can be used to address this problem in stochastic computational mechanics. The widely used Monte Carlo simulation requires significant CPU power, particularly when probability density functions (PDFs) of reduced von Mises stress and deflections are required. Therefore, the 2nd-order stochastic perturbation method [9] was initially used to determine the nonlinear probabilistic response of a structure with internal structural defects. It has to be underlined that the determination of higher-order probabilistic characteristics is not allowed in this approach, and its insufficiency is obvious when the input statistical dispersion is large. To avoid these inconveniences, the iterative generalized stochastic perturbation method (IP), implemented in conjunction with the FEM—ABAQUS System, is employed to investigate the impact of structural defects on the behavior of constructional steel according to the Gurson–Tvergaard–Needleman (GTN) theorem. The classical tension test of the cylindrical steel element [10] was employed in this case, given the extensive research on this topic. The iterative stochastic perturbation method, which utilizes a Taylor expansion up to the 10th order and the Weighted Least Squares Method (WLSM) to define polynomial responses, has been conducted. Deformation problems involving geometric and material nonlinearities have been studied.

A failure danger can be expressed in terms of its probability, but its use is instead justified by scientific work. On the other hand, knowledge of probabilistic relative entropy can be the solution to these problems. The key assumption of the model presented here is that the porosity at the beginning of the analysis follows a Gaussian distribution (according to the Central Limit Theorem [11]), with a mean and coefficient of variation indicating a large spread. According to refs. [12,13], the micro-void ratio typically ranges from 0 to 0.0024 for constructional carbon steel; the value, of course, indicates a complete lack of micro-voids (a continuum material). According to Eurocode and the literature, the size and location of these structural imperfections can vary, which is the main reason we randomized the initial void volume fraction parameter f_0_. Various studies and research [14] have determined the range of this coefficient. This paper aims to assess reliability coefficients β based on the Bhattacharyya relative entropy and compare them with those obtained using the FORM technique. This paper extends the authors’ previous research on safety assessment using relative entropy theory.

## 2. Governing Equations

### 2.1. Nonlinear Solid Mechanics Equations

**Figure 1:**
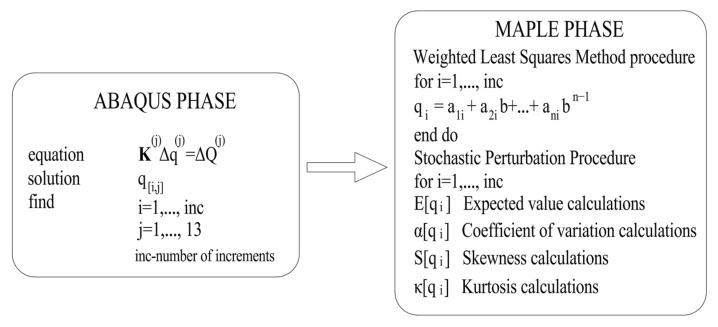
Stochastic Finite Element Method procedure scheme.

The following boundary value problem of nonlinear solid mechanics is considered [15]:

(1) $$\Delta \sigma_{kl,l}+\rho \Delta f_{k}=0;\;x\in \Omega$$

(2) $$\Delta {\tilde{\sigma }}_{kl}=C_{klmn}\Delta \varepsilon_{mn};\;x\in \Omega$$

(3) $$\Delta \varepsilon_{mn}=\frac{1}{2}[\Delta u_{k,l}+\Delta u_{l,k}+u_{i,k}\Delta u_{i,l}+\Delta u_{i,k}u_{i,l}+\Delta u_{i,k}\Delta u_{i,l}];\;x\in \Omega$$

for *i*, *j*, *k*, *l* = 1, 2, 3 with the given boundary conditions:

(4) $$\Delta \sigma_{\bar{k}l}n_{l}=\Delta t_{\bar{k}};\;x\in \partial \;\Omega_{\sigma },\;\bar{k}=1,2,3$$

(5) $$\Delta u_{\hat{k}}=\Delta {\hat{u}}_{\hat{k}};\;x\in \partial \;\Omega_{u},\;\hat{k}=1,2,3$$

where $u_{k}\left(x\right)$ means the displacement vector, $\varepsilon_{kl}\left(x\right)$ the strain tensor, and the stress tensor $\sigma_{kl}\left(x\right)$, according to von Neumann and Dirichlet boundary conditions (Equations (4) and (5)). $C_{klmn}$ denotes the constitutive tensor here referred to as the porosity problem [16]. Its solution is based on minimizing the potential energy functional with respect to the displacement vector, a well-known approach in the Finite Element Method discretization for nonlinear problems [17]. The probabilistic method is based on a deterministic series of solutions of the iterative FEM equation:

(6) $$K^{\left(j\right)}{\Delta q}^{\left(j\right)}={\Delta Q}^{\left(j\right)}$$

where **K** is the stiffness matrix, ΔQ represents the nodal loads’ increments vector, and Δq is the displacements vector increments. The upper indices *j* correspond to the current FEM test number. Equation (6) has been solved in the ABAQUS system for eleven discrete realizations of each random variable (ABAQUS phase), and then the MAPLE phase begins. Based on those FEM test results, a polynomial response approximation has been found with the usage of the Weighted Least Squares Method (WLSM). Further determination of the probabilistic moments and characteristics (expected value, standard deviation, skewness, kurtosis) proceeds, thanks to the symbolic derivation of all partial derivatives with respect to the given input random variable. Figure 1 presents a detailed flowchart of the procedure.

### 2.2. GTN Material Model Equations

The classical Gurson porous material model [10] assumes that the plastic potential function is based on the volume fraction *f*. It underwent numerous modifications, and one of the most significant changes introduced by Tvergaard [18] was the coefficients $q_{i}$, later referred to as the Tvergaard parameters, which enhance specific plastic properties. Values of $q_{i}$ taken in this paper are typical for metallic materials and equal to $q_{1}=1.5,\;q_{2}=1.0,\;q_{3}=q_{1}^{2}=1.5^{2}=2.25$. It has to be underlined that the Tvergaard coefficients affect the Young’s modulus and yield stress, which are the main steel parameters. Including those parameters and later modifications, the Gurson–Tvergaard–Needleman yield condition is given as follows:

(7) $$\Phi ={\left(\frac{\sigma_{e}}{\sigma_{0}}\right)}^{2}+2q_{1}f*\cosh\left(-q_{2}\frac{3\sigma_{m}}{2\sigma_{0}}\right)-(1+q_{3}f{*}^{2})=0$$

where $\sigma_{e}=\frac{1}{\sqrt{2}}\sqrt{{\left(\sigma_{1}-\sigma_{2}\right)}^{2}+{\left(\sigma_{2}-\sigma_{3}\right)}^{2}+{\left(\sigma_{1}-\sigma_{3}\right)}^{2}}$ is the reduced stress according to the Huber–Mises–Hencky theorem, $\sigma_{0}$ is the yield point, $\sigma_{m}$ is the hydrostatic stress, f* represents the actual void volume fraction (VVF), and it is calculated according to the following formula [19]:

(8) $$f*=\left\{\begin{array}{lll} f & \mathrm{for} & f\leq f_{c}\\ f_{c}+\frac{{\bar{f}}_{F}-f_{c}}{f_{F}-f_{c}}\left(f-f_{c}\right) & \mathrm{for} & f_{c}<f<f_{F}\\ {\bar{f}}_{F} & \mathrm{for} & f\geq f_{F} \end{array}\right.$$

where *f_c_* is the critical voids volume fraction, which is related to the beginning of their joining, and $f_{F}$ is the critical void volume fraction, understood as a material damage, and ${\bar{f}}_{F}$ is the coefficient which says how fast new voids f* appear according to (9):

(9) $${\bar{f}}_{F}=\left(q_{1}+\sqrt{{q_{1}}^{2}-q_{3}}\right)/q_{3}.$$

Gurson’s original parameter *f* was modified to describe the phenomena that occur if the void volume fraction *f* is greater than *f_c_*.

**Figure 2:**
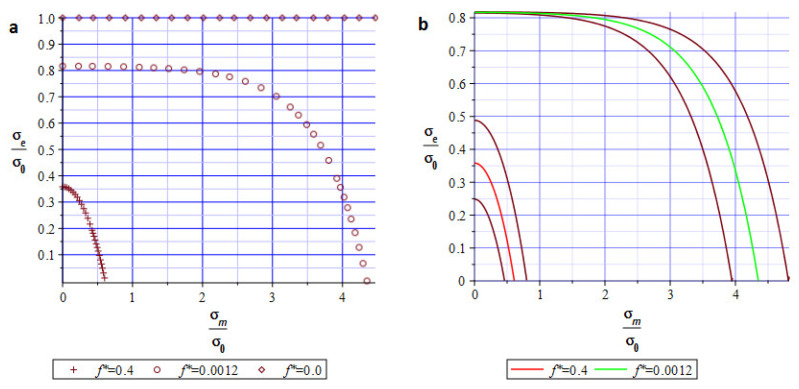
(**a**) Pressure-dependent yield function for different porosity coefficient *f_v_*. (**b**) Yield surface for f* = 0.001 and f* = 0.4 with its random dispersion.

Figure 2a shows yield surface distributions for the uniaxial stress state related to the numerical experiment presented in the following section. Figure 2b presents the expansion of the previous one, incorporating the dispersion of the random parameter f*. The red and green middle lines represent the expected values of the void volume fraction. The adjacent brown lines define the dispersion of the random variable. Both figures show strong relationships and dependencies among volume fraction, effective stress (according to the HMN hypothesis), yield point, and hydrostatic pressure. An idea for randomizing porosity is not new and has been implemented using homogenization theory, for instance, refs. [20,21].

The experiments and computational tests had been conducted to determine the values of those coefficients. Let us assume that the volume fraction of voids existing in the material is set as $f_{0}$, which corresponds to porosity at the beginning of the process. It means that before deformation starts and $f*=f_{0}$ [22,23]. Moreover, implies that the material has no voids and that the Mises yield conditions $f_{0}=1$ are valid; this means that the material is completely voided and has no stress resistance capacity. From the probabilistic point of view, it has to be underlined that the growth of voids is described [24]:

(10) $$\dot{f}={\dot{f}}_{gr}+{\dot{f}}_{nucl}=\left(1-f\right){\dot{\varepsilon }}^{pl}:I+\frac{f_{N}}{s_{N}\sqrt{2\pi }}\exp\left[-\frac{1}{2}{\left(\frac{\varepsilon_{em}^{pl}-\varepsilon_{N}}{s_{N}}\right)}^{2}\right]\cdot {\dot{\varepsilon }}_{em}^{pl}$$

where the following notation applies: $\dot{f}$ denotes a growth of the void volume fraction, ${\dot{f}}_{gr}$ stands for the growth of the void volume fraction of voids existing in the material, ${\dot{f}}_{nucl}$ is a growth rate of the void volume fraction of voids due to their nucleation, and $f_{N}$ means the growth of the void volume fraction nucleated. Furthermore, $s_{N}$ is the standard deviation of the void nucleation strain, ${\dot{\varepsilon }}^{pl}$ is the tensor of plastic strain rate increase, I is the second-order tensor defining the growth rate of plastic strain [25], $\varepsilon_{N}$ is the mean void nucleation strain, $\varepsilon_{em}^{pl}$ is the equivalent plastic strain, whereas ${\dot{\varepsilon }}_{em}^{pl}$ is the equivalent plastic strain growth rate. Given that many of these parameters are derived from experimental analysis, their statistical treatment seems natural. The same concerns apply to material micro-voids, which can be detected using image analysis and quantified using statistical parameters such as radius and frequency. The methodology and application of the relative entropy shown in this paper are a development of the authors’ previous research [26], extended by the GTN porous material model. Moreover, in [27], a GTN material model for locally functionally graded materials is presented.

### 2.3. Uncertainty Analysis with Relative Entropy

The nucleation process is probabilistic. Because the parameter $f_{0}$ is set as the input Gaussian random variable here, the plastic surface is no longer an ellipsoid. Engineering intuition and experience suggest that micro-voids are distributed disorderly throughout the cross-section and along the length of the specimen.

Due to the limitations of the First-Order Reliability Method, an alternative is frequently sought. One of the most significant disadvantages of the FORM methodology is the requirement that limit functions have to be linear. In this paper, the limits of highly nonlinear stress, deflection, and displacement state functions are considered. That is the reason why we propose an algorithm for safety assessment based on relative entropy. Its fundamental novelty lies in demonstrating that the reliability assessment has not been established for metal structures using the relative entropy apparatus, specifically when employing the GTN theory for solids.

From the probabilistic point of view, entropy is the divergence between two different probability distributions [28]. The distance between the admissible structural effort *R* and the extreme structural effort *E* can be a measure of the structural safety. The relative entropy proposed here requires no information about the distributions of *E* and *R*, other than what the FORM technique provides, which is efficient to use assuming those distributions are Gaussian. Relative entropy is a measure of the randomness of the phenomenon being studied. One relative entropy plot can replace expectations, variance, skewness, and kurtosis, providing the same information about the distribution.

Equation (11) presents the reliability index according to the First-Order Reliability Method, which is also used in Eurocode standards [29].

(11) $$\beta_{FORM}=\frac{E\left[E\right]-E\left[R\right]}{\sqrt{Var\left(R\right)-Var\left(E\right)}}.$$

According to Bhattacharyya’s theory [30], the distance from *E* (effective design) to *R* (resistance design) can be given as follows:

(12) $$H_{B}\left(R,E\right)=\frac{1}{4}\frac{\left(E\left[R\right]-E\left(E\right)\right)}{\sigma^{2}\left(R\right)+\sigma^{2}\left(E\right)}+\frac{1}{2}\ln\left(\frac{\sigma^{2}\left(R\right)+\sigma^{2}\left(E\right)}{2\sigma \left(R\right)\sigma \left(E\right)}\right).$$

The derivation and use of formula (12) can be found in reference [31]. We aim to use the well-established reliability index intervals specified in the design codes; due to the similarity of the first two components in Equations (11) and (12), we proposed rescaling by extracting the reliability index from the Bhattacharyya distance.

(13) $$\beta =\frac{1}{2}\sqrt{H_{B}\left(R,E\right)}$$

Without rescaling, using relative entropy is more complex, and the results may be overestimated. If the uncertainty levels in *R* and *E* are similar, this approach reduces to the FORM approach. However, it should be noted that this approach is based on a relative entropy technique (14) that uses a general formula for any two probability distributions of the variables *R* and *E* (for linear and nonlinear state functions).

(14) $$H_{B}\left(p\left(R\right),p\left(E\right)\right)=\int_{-\infty }^{+\infty }{\left\{p\left(R\right)\left(x\right)p\left(E\right)\left(X\right)\right\}}^{1/2}dx$$

Previous studies on construction safety are referenced for structural elastic–static and elastic–dynamic design problems, confirming their effectiveness. Models given by Kullback & Leibler [32] have been contrasted here, and the following holds:

(15) $$H_{K-L}\left(p\left(R\right),p\left(E\right)\right)=-\int_{-\infty }^{+\infty }p\left(R\right)\left(x\right)\log\left(p\left(E\right)\left(x\right)\right)dx+\int_{-\infty }^{+\infty }p\left(E\right)\left(x\right)\log\left(p\left(R\right)\left(x\right)\right)dx$$

The entropies given above will be used when the normal distribution is defined as non-truncated. It may result in a small modeling error in specific engineering problems where structural parameters exhibit a truncated character.

## 3. Numerical Simulations

### 3.1. The First Case Study of an Extended Steel Cylindrical Bar

**Figure 3:**
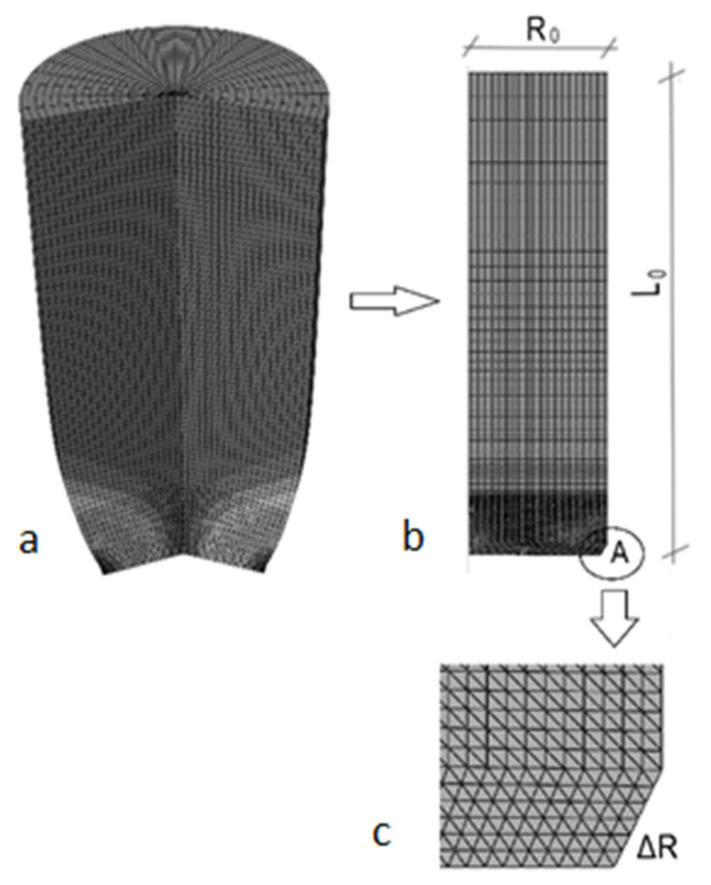
Cylindrical bar, its specimen, and the discretization scheme [26]. (**a**) Initial geometry of the specimen. (**b**) Transformation from the bar to the plane sample. Its discretization and dimensions: length L_0_ = 40 mm, radius R_0_ = 10 mm. Specimen meshing: bottom part with minimal CAX3 elements (three-node linear axisymmetric triangular finite elements). Rest—CAX4R (four-node quadrilateral elements with reduced integration). (**c**) Notch Δ*R* = 0.5 × 1.0 mm.

Verification of how structural defects in carbon steel affect its behavior is under consideration in this initial study. Incremental responses were conducted using classical, deterministic, quasi-static steel-specimen extension. Numerous phenomena can influence the behavior of steel structural elements under significant tensile stresses. It should be noted that the modeling error originates from coalescence, imperfection growth, and nucleation disorder. The microdefects are a few times smaller than typical bolt holes in steel structures, but their proliferation, increased diameter, and greater number may lead to a similar effect and material failure. Figure 3 shows the geometry and mesh of the specimen, and that, due to its horizontal and vertical symmetry, only a slice of the round bar has been used for computational purposes. The radius of the specimen equals $R_{0}=10\;\mathrm{mm}$ and its length is set $L_{0}=40\;\mathrm{mm}$ to, so that their ratio is $L_{0}{/R}_{0}=4$, based on previous numerical research [10]. Detail “A” shows a tiny cut (ΔR=0.5×1.0 mm) in the bottom-right corner to ensure that necking starts in the middle of the specimen. One of the most popular S235JR carbon steel grades has been taken for this analysis. Due to horizontal and vertical symmetry, kinematic boundary conditions are provided $u_{x}=0$ on the left edge and $u_{y}=0$ the bottom. To ensure sample elongation, a kinematic forced displacement of the top edge (4.0 mm) has been applied. The presented specimen was divided into two finite element types. Part I (the process zone) is near the bottom edge, where necking is expected. 2548 CAX3 elements have been used here (three-node linear axisymmetric triangular finite elements). According to ref. [10], the triangle’s side is 0.2 mm. It is the characteristic length typical for S235JR steel (cell model). For the discretization of the other part of the specimen, 2156 CAX4R (four-node quadrilateral elements with reduced integration) FEM elements were used. The horizontal size of these elements is 0.2 mm to match the previous one, and the vertical side of the rectangle varies from 0.2 to 2.0 mm, increasing from the bottom to the top of the specimen. Such a differentiation of the mesh scheme yields better results than the classical one and reduces the number of FEM elements. This approach provides a more detailed description of the actual phenomena and the evolution of microdamage, which is crucial here [33].

**Figure 4:**
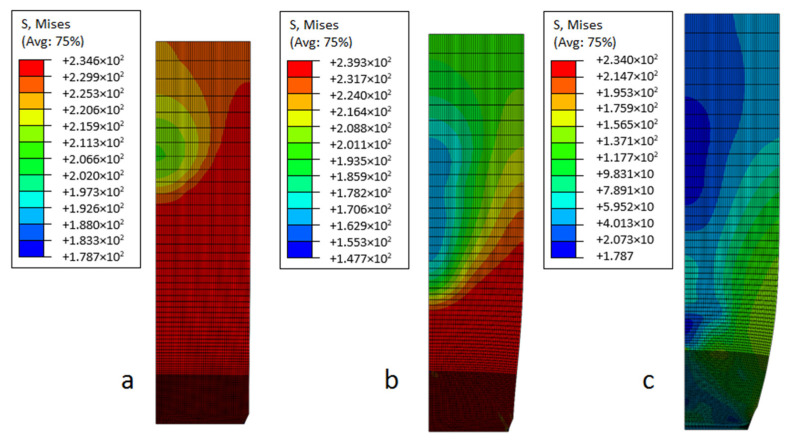
Von Mises stress distribution (MPa) for initial micro-voids [26]. (**a**) *f*_0_ = 0.0000, (**b**) *f*_0_ = 0.0012, and (**c**) *f_0_* = 0.0024.

In this model, Young’s modulus *E* = 210 GPa, Poisson’s ratio ν = 0.3 and yield point $\sigma_{pl}=235\;\mathrm{MPa}$ have been used. Moreover, the Tvergaard coefficients mentioned in the Introduction para-graph are set to $q_{1}=1.5$, $q_{2}=1.0$ and $q_{3}=q_{1}^{2}=1.5^{2}=2.25.$ The volume fraction of nucleated voids here is $f_{N}=0.04,$ while the average nucleation strain is assumed to be $\varepsilon_{N}=0.3$ and the standard deviation of the nucleation strain is $s_{N}=0.10.$ The initial volume fraction of voids existing in the material $f_{0}$ is one of the most critical parameters in this paper, and has been taken as a Gaussian random variable. According to ref. [19], its dispersion of 0.0000–0.0024 is typical for constructional steel. A step of this parameter is assumed to be equal to 0.0002. Figure 4 shows the reduced von Mises stresses distribution obtained for three different initial microdefects $f_{0}$. As it can be seen, the shapes of those plots are pretty similar, which is why the micro-voids assume the same size but its number is different. On the other hand, the stress distribution is not equal for all values of $f_{0}$. The most significant reductions in stress occur when there are no initial defects $f_{0}=0.0000$ (material continuum). Before nucleation, the reduced von Mises stresses are nearly constant and close to the plastic limit. Moreover, significant variations in values slightly below the yield strength are observed and influenced by micropores.

Further computational experiments begin with determining polynomial functions [34] that represent the structure’s local response. Table 1 presents the polynomial order, correlation indices, and RMS errors resulting from the Weighted Least Squares Method for one of the 130 displacement polynomial function fits. It was conducted for horizontal displacements and reduced von Mises stresses, taken from the bottom-right corner area (Figure 3). It can be seen that an 8th-order polynomial provides the best approximation.

**Figure 5:**
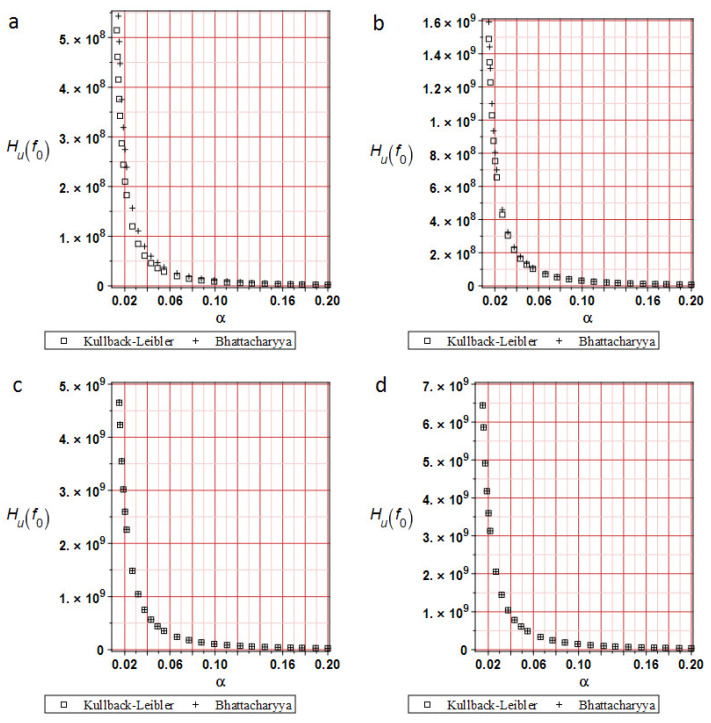
Relative entropies comparison for the horizontal displacements (**a**) at 20% of the analysis progress, (**b**) 50% of the analysis progress, (**c**) 70% of the analysis progress, and (**d**) 100% of the analysis progress.

Figure 5 shows a comparison of relative entropies calculated using different methods at analysis progress levels of 20%, 50%, 70%, and 100%. Typically, in reliability analysis, relative entropy decays exponentially across models, depending on the level of input uncertainty. This simulation demonstrates that the distribution of relative entropy depends on the input coefficient of variation α. At the very beginning of the deformation process, the Kullback–Leibler relative entropy distribution yields slightly fewer values than the Bhattacharyya approach. At about 70% of the analysis, both techniques yield the same values and distributions. A remarkable result is that the extreme values of relative entropies decrease as the deformation process progresses, which is consistent with experimental observations [35]—the expectation of the extreme displacement approaches its admissible counterpart. Compatibility between the two approaches allows comparison of the reliability indices *β* calculated using the FORM and Bhattacharyya techniques, due to their component similarities (Equations (11) and (12)).

**Figure 6:**
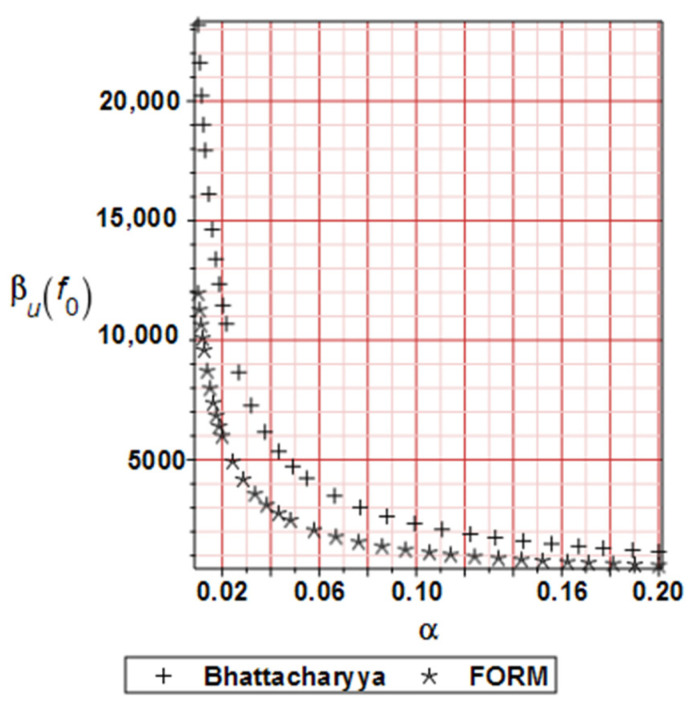
Numerical example 1-Reliability index based on FORM and Bhattacharyya relative entropy comparison.

Figure 6 illustrates the distribution of the reliability index for horizontal displacements, based on the FORM and Bhattacharayya relative entropy. The curvature of those plots deserves some attention. Nevertheless, the reliability indices exhibit a swift decay, accompanied by an additional increase in the input uncertainty of the micro-voids volumetric ratio. Additionally, the numerical values of these indices decrease as the deformation process progresses for the same reason mentioned above.

### 3.2. The Second Numerical Illustration—The Both Ends Fixed Beam

**Figure 7:**
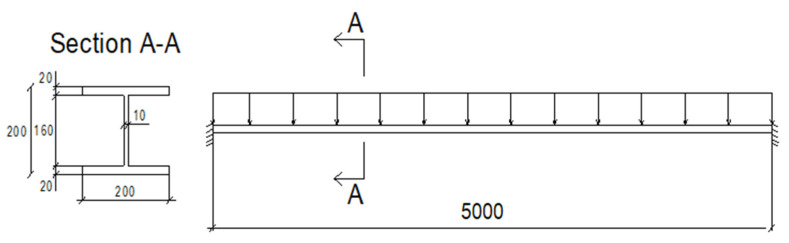
Geometrical data of the beam structure.

Another numerical experiment is a 5 m-span steel I-beam. Displacements and rotation degree of freedom equal 0 at its ends. Figure 7 shows its length, boundary conditions, load distribution, and cross-section dimensions.

**Figure 8:**
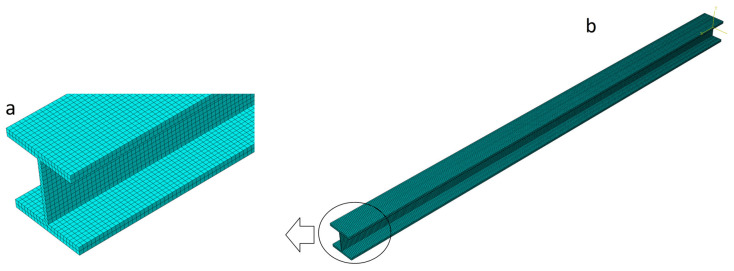
Discretization of the beam. (**a**) Detailed cross-section discretization, (**b**) 38,000 C3D8R-hourglass control with reduced-integration linear brick FEM elements.

A total of 38,000 C3D8R (hourglass-controlled, reduced-integration linear brick) finite elements were used in this process (Figure 8). The external load was distributed to the top flange with a magnitude of 7 kN/m. The full Newton procedure was conducted with the following parameters: the initial increment size was set to 0.0001, and the minimum and maximum values were set to 0.1 and 1.0, respectively. As it was assumed previously, the initial porosity f_0_ was taken as a Gaussian random variable with its range [0.0000, 0.0024] and its expected value $E\left[f_{0}\right]=0.0012;$ the coefficient of variation (CoV) of it is assumed to belong to the interval [0.00, 0.20]. The porous metal plasticity model incorporates Tvergaard coefficients, adopted here after the initial study, with *q*_1_ = 1.5, *q*_2_ = 1, and *q*_3_ = 2.25, representing the volume fraction of nucleated voids, the average strain at the appearance of new voids, and its standard deviation, $s_{N}=0.10$ [36,37]. Mechanical steel properties in the ABAQUS system are the same as those in the first example. A Young modulus of *E* = 210 GPa, a yield point of $\sigma_{pl}=235\;\mathrm{MPa}$, and also Poisson’s ratio ν = 0.3 [38]. Table 2 presents polynomial order, correlation indexes, and RMS Errors as a result of the usage of the Weighted Least Squares Method for one of the 130 deflection polynomial functions. Similar to the previous numerical simulation, an 8th-order polynomial provides the best approximation accuracy.

**Figure 9:**
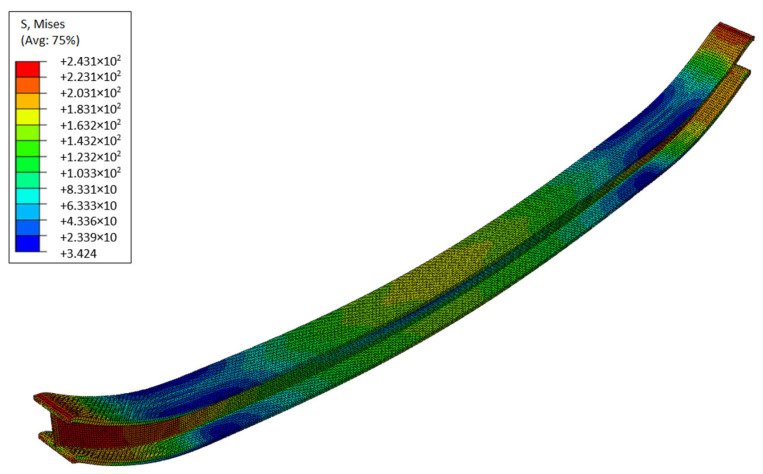
Resulting plots for the reduced von Mises stress [26].

Figure 9 shows the resulting von Mises stress map at the end of the analysis, calculated for the mean initial micro-void volume fraction, *f*_0_ = 0.0012 [39]. Surely, the extreme stresses appear at the ends of the beam where displacements and rotations of the sections are fixed. On the other hand, the most significant deformations occur at the midpoint of the beam.

Due to porosity phenomena, it should be noticed that the shape of the deformed span is not as uniform as for the continuum material model (without defects). For the same reason, large deformations are observed at the restrained ends, where the current void volume fraction VVF obtains its highest values. Therefore, limit-state analysis, which is crucial for reliability research, should be based on a static failure scenario with two plastic joints. Ribs welded to an I-profile beam in the supporting area can reduce the effects of porosity, thereby increasing durability and safety.

**Figure 10:**
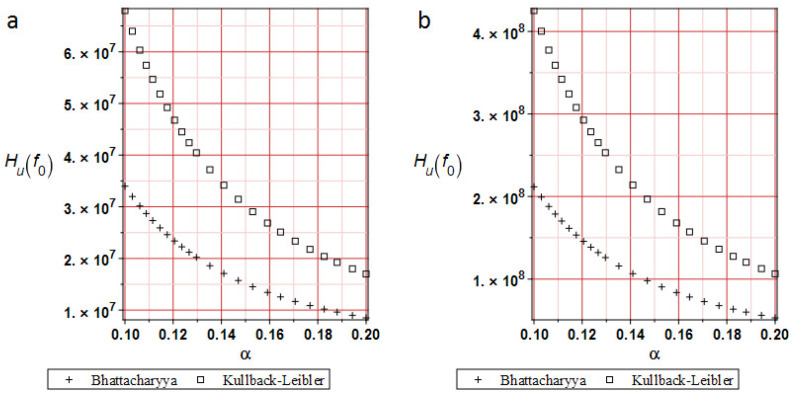

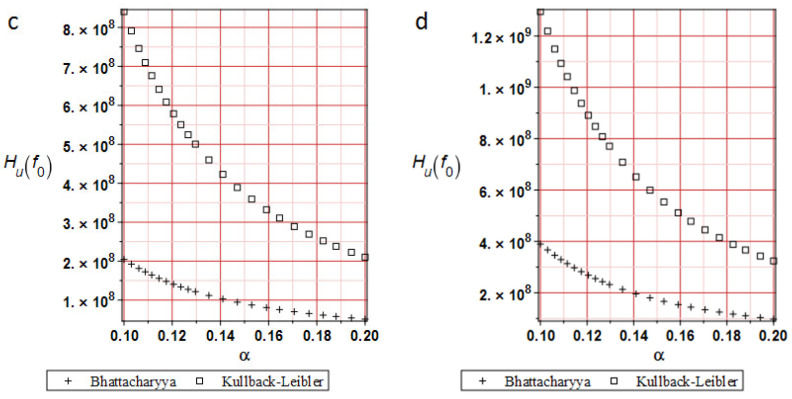
Relative entropies comparison for the deflections. (**a**) at 20% of the analysis progress, (**b**) at 50% of the analysis progress, (**c**) 80% of the analysis progress, and (**d**) 100% of the analysis progress.

Figure 10 shows a comparison of relative entropies at 20%, 50%, 70%, and 100% of analysis progress. These results indicate that relative entropy distributions depend on the input coefficient of variation α. Moreover, the Bhattacharyya entropies are smaller than those calculated using the Kullback–Leibler theorem, and their divergence is slightly larger at the end of the analysis. It may be caused by the size and type of the FEM element used. For the first test in the lower part of the specimen, FEM elements smaller than 1 mm have been used. In this case, 8-noded 10 mm side size cubic FEM elements have been taken. Using 1 mm cubic FEM elements would lead to a significant increase in computation time or even convergence problems. On the other hand, the entropy distributions have quite similar plots. A larger input CoV α yields smaller entropy values.

**Figure 11:**
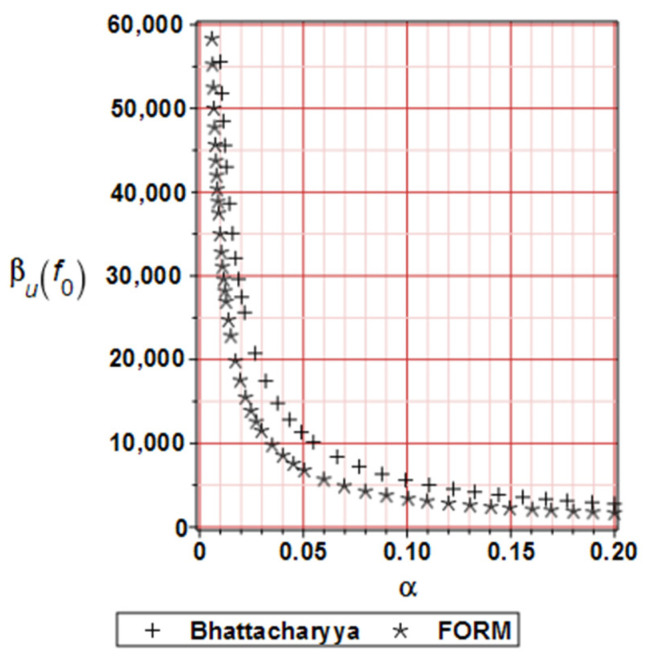
Numerical example 2-Reliability index based on FORM and Bhattacharyya relative entropy comparison.

Figure 11 illustrates the distribution of the reliability index for the deflections calculated using the FORM and Bhattacharyya relative entropy approaches as before. It is essential to emphasize that the curvatures and values are pretty similar, which is the primary conclusion here. It is evident that the relative entropy index may sometimes yield less favorable results than the FORM approach [40], which is usually treated as the reference solution. This is due to the high nonlinearity of the limit function in the given case study.

## 4. Conclusions

(1) The relative entropy determination during probabilistic nonlinear deformation of metals with internal porosity has been presented in this paper using various models pertinent to stochastic mechanics. Their exponential decay, together with increasing uncertainty in micropores’ geometrical parameters and the advancing deformation process, has been shown and discussed here. Additionally, the reliability index calculated using relative entropy is very close to that obtained with the classical First-Order Reliability Method (FORM). Relative entropy safety assessment is more flexible than FORM, Second Order Reliability Method (SORM), and Weibull-Second Order Third Moment (W-SOTM), and does not require the assumption of the Gaussian distribution of state functions. Relative entropy is a real-valued function that shows random chaos on a single graph, rather than a series of plots with different parameters.
(2) It has been demonstrated that random void volume fraction has a large influence on the yield surface and yield function distribution, and its random character should be taken into account in the reliability analyses of steel structures with porosity. The Stochastic Finite Element Method (SFEM), implemented using the iterative generalized stochastic perturbation method, can be used efficiently to determine the stresses and deformations in metals with statistically distributed geometric micropores. Such imperfections are modeled here using the Gurson–Tvergaard–Needleman porous material model. It enables relatively short computations of up to fourth-order probabilistic moments and characteristics in deforming metals, including various types of steel, a variety of aluminum alloys, and copper. A very convenient aspect demonstrated here is the capability of the hybrid implementation of the FEM software ABAQUS with a computer algebra system (such as MAPLE 2025) for such multiscale material models.
(3) The proposed multiscale constitutive model of the porous metal should be extended in future studies to large thermomechanical deformations, including pore coalescence and pore growth, not only due to mechanical boundary conditions but also due to heating (or freezing) of the material. This model should include temperature-dependent variations in both material parameters and pore size and number. Another interesting topic would be the automation of polynomial and non-polynomial response function fitting, where machine learning algorithms could efficiently replace the Weighted Least Squares Method presented above.

## Figures and Tables

**Table 1:** Displacements polynomial order, correlation index and RMS error.

Polynomial Order	Correlation Index	RMS Error
2	0.9954721794	0.825882
3	0.9986646659	0.448865
4	0.998884485	0.410281
5	0.9992027318	0.346882
6	0.9997217842	0.204971
7	0.9997218748	0.204915
8 *	0.9998935197	0.129167
9	0.5496160627	328.245
10	0.9998334416	0.158824

* The best approximation order.

**Table 2:** Deflections polynomial order, correlation index and RMS error.

Polynomial Order	Correlation Index	RMS Error
2	0.9953634143	0.00157754
3	0.9928809534	0.0105956
4	0.9987766101	0.000811033
5	0.9995234094	0.000506300
6	0.9998497709	0.000284298
7	0.9999619796	0.000143136
8 *	0.9999925247	0.0000649093
9	0.4581667499	1.51560
10	0.9999327601	0.000212479

* The best approximation order

## Data Availability

The original contributions presented in this study are included in the article. Further inquiries can be directed to the corresponding author.
